# Freehand Pedicle Screw Placement Using a Universal Entry Point and Sagittal and Axial Trajectory for All Subaxial Cervical, Thoracic and Lumbosacral Spines

**DOI:** 10.1111/os.12599

**Published:** 2019-12-11

**Authors:** Zheng‐feng Zhang

**Affiliations:** ^1^ Department of Orthopaedics Xinqiao Hospital, Third Military Medical University Chongqing China

**Keywords:** Freehand technique, Lumbar, Pedicle screws, Subaxial cervical, Thoracic

## Abstract

**Objective:**

Existing techniques of freehand pedicle screw placement primarily focus on various entry points with or without axial trajectory. The objective of this paper is to propose a universal entry point and sagittal and axial trajectory for all subaxial cervical, thoracic and lumbosacral spines freehand pedicle screw placements, and to report the results from a single‐surgeon clinical experience with freehand pedicle screw placement.

**Methods:**

Two spine vertebrae specimens and 20 cases of three‐dimensional (3D) reconstructions of spine CT images were used for observation of the entry point and sagittal and axial trajectory. The author retrospectively reviewed a total of 610 consecutive patients who underwent open, freehand pedicle screw fixation using a universal entry point and sagittal and axial trajectory for all subaxial cervical, thoracic and lumbosacral spine placements, during an 8‐year period from January 2010 to December 2017. The junction of the lateral margin of the superior articulating process and the transverse process for the thoracic and lumbosacral spines, or lateral mass for the subaxial cervical spine, was determined. The entry point was chosen at 1 mm, 2 mm, and 3 mm (2 mm on average) caudally and medially to this junction for subaxial cervical, thoracic and lumbosacral spines placements, respectively. Both sagittal and axial trajectories were perpendicular to the sagittal and axial planes of the laminae of the isthmus. Among them, 68 patients underwent postoperative computed tomography (CT) scans, including 26 cervical cases, 19 scoliosis thoracic cases, 10 non‐scoliosis thoracic cases, 8 lumbar cases, and 5 sacral cases. Placements of pedicle screws were assessed using CT data and outcome‐based classifications systems.

**Results:**

After placing the iron scurf at the junction of the lateral margin of the superior articulating process and the transverse process, the present universal entry point was located at 1 o'clock or 11 o'clock of the pedicle's axial view. After inserting the 2.5 mm Gram needle or the pedicle virtual pin tracts according to the entry point and sagittal and axial trajectory described above, the presented trajectory was located in the pedicle's axial trajectory as in the described technique. A total of 766 pedicle screws were placed in 68 CT scan patients with a 99% accuracy rate in the non‐kyphoscoliosis group and 92% in the kyphoscoliosis group.

**Conclusions:**

Freehand pedicle screw placement based on the universal entry point and sagittal and axial trajectory for all subaxial cervical, thoracic and lumbosacral spines can be performed with acceptable safety and accuracy.

## Introduction

Pedicle screw fixation is the preferred modality throughout the vertebral column because of its proven effectiveness in stabilizing all three columns of the spine. This advantage has been translated to superior clinical results in certain cases of trauma, instability, deformity, and neoplastic destruction. Therefore, it has become popular during the past decade, first in applications involving the lumbar spine and subsequently in thoracic and cervical spine surgery. However, pedicle screws remain technically demanding to place, particularly in the cervical and thoracic region because of the smaller size and more complex morphology of pedicles. Additionally, complications related to the use of screws are potentially serious, including: screw misplacement, pedicle fracture, screw rupture, bending or loosening, vertebral canal violation, dural tear, vascular or visceral problems, and postoperative neurologic symptoms or pain. Because of this, the freehand pedicle screw insertion techniques and image‐guided techniques have been widely developed to guide placement.

Freehand pedicle screw insertion technique relies on tactile feedback of the surgeon and the use of anatomical marks to determine correct screw entry point, without the aid of intraoperative image‐guided systems or explorative laminectomy, with no or limited use of intra‐operative fluoroscopy. While the freehand methods are effective and widely employed^1^, ^2^, ^3^, ^4^, ^5^, ^6^, ^7^, ^8^, ^9^, ^10^, ^11^, ^12^, ^13^, the main shortcoming of freehand technique is the rather long learning curve, as the successful placement of the screws depends entirely on surgeon ability and great experience is demanded to obtain good results. Because of these concerns, several image‐guided techniques have been used to increase the ease and accuracy of pedicle screw placement, including the use of intraoperative fluoroscopy, intraoperative CT, image‐assisted navigation, robotic guidance system, and impedance‐measuring probe. Although these expensive pieces of equipment and techniques slightly increase placement accuracy, these methods have increased cost and relied on technology which is still not without flaws. These modalities have also been associated with increased operative time and radiation exposure to both the patient and the surgeon^14^, ^15^. This radiation exposure is not without risk, especially for surgeons who perform numerous surgeries over the course of a career. Despite the progress in technology, some surgeons are more comfortable with the classical freehand technique for achieving good results. A safe, reproducible, and reliable practice of freehand pedicle screw placement is ideal for reducing intraoperative radiation exposure, reducing the overall operative time, and streamlining the teaching process for surgical trainees.

As the free‐hand pedicle screw placement technique relies solely on anatomy, existing techniques primarily focus on various entry points with or without medial angulations (axial trajectory)^1^, ^2^, ^3^, ^4^, ^5^, ^6^, ^7^, ^8^, ^9^, ^10^, ^11^, ^12^, ^13^. For example, the most popular approach places the lumbar entry point at the intersection of the transverse process and the pars interarticularis. Various quantitative medial angulations have been described for all subaxial cervical, thoracic and lumbosacral sections of the spine according to different segments and entry points. Sagittal trajectories have also been defined to be orthogonal to the curvature of the dorsal spine at that level or to the superior endplate of the corresponding vertebral body. However, most of these studies describe different entry points at different levels as well as different insertion techniques. In addition, the transverse process is involved, and the superior facet is used as a landmark. The situation changes in the case of a deformed spine as the transverse process and/or the superior articular process may be abnormal in shape and size due to rotation and deformity.

Although different entry points at different levels as well as different trajectories have been employed, neither universal entry point nor consistent methods to determine cranial‐caudal (sagittal) trajectory based on anatomic landmarks have been described^1^, ^2^, ^3^, ^4^, ^5^, ^6^, ^7^, ^8^, ^9^, ^10^, ^11^, ^12^, ^13^. Based on the junction of the lateral margin of the superior articulating process and the transverse process or lateral mass, and the laminae of the isthmus developing from the same vertebral arches in the embryologic stage, the author developed a freehand pedicle screw placement technique of universal entry point and sagittal and axial trajectory. This entry point is easy to explore and identify without the influence of ossification and is easier to visualize intraoperatively than the anamnesis of traditional corresponding angulars for different levels; it is also effective with kyphoscoliosis deformities. To evaluate this possibility, we have used spine vertebrae specimens and three‐dimensional (3D) reconstructions of spine CT images and attempted to determine the present universal entry point location and sagittal and axial trajectory. We have also assessed the placements of pedicle screws with non‐kyphoscoliosis and kyphoscoliosis patients in the subaxial cervical, thoracic and lumbosacral spines. The purpose of this study was: (i) to propose a universal entry point and sagittal and axial trajectory for all subaxial cervical, thoracic and lumbosacral spine freehand pedicle screw placements; (ii) to determine the accuracy of this freehand technique by a single‐surgeon clinical experience; and (iii) to employ universal freehand technique, which is easier to learn, teach, and adopt.

## Patients and Methods

### *Entry Point and Sagittal and Axial Trajectory*

**Figure 1:**
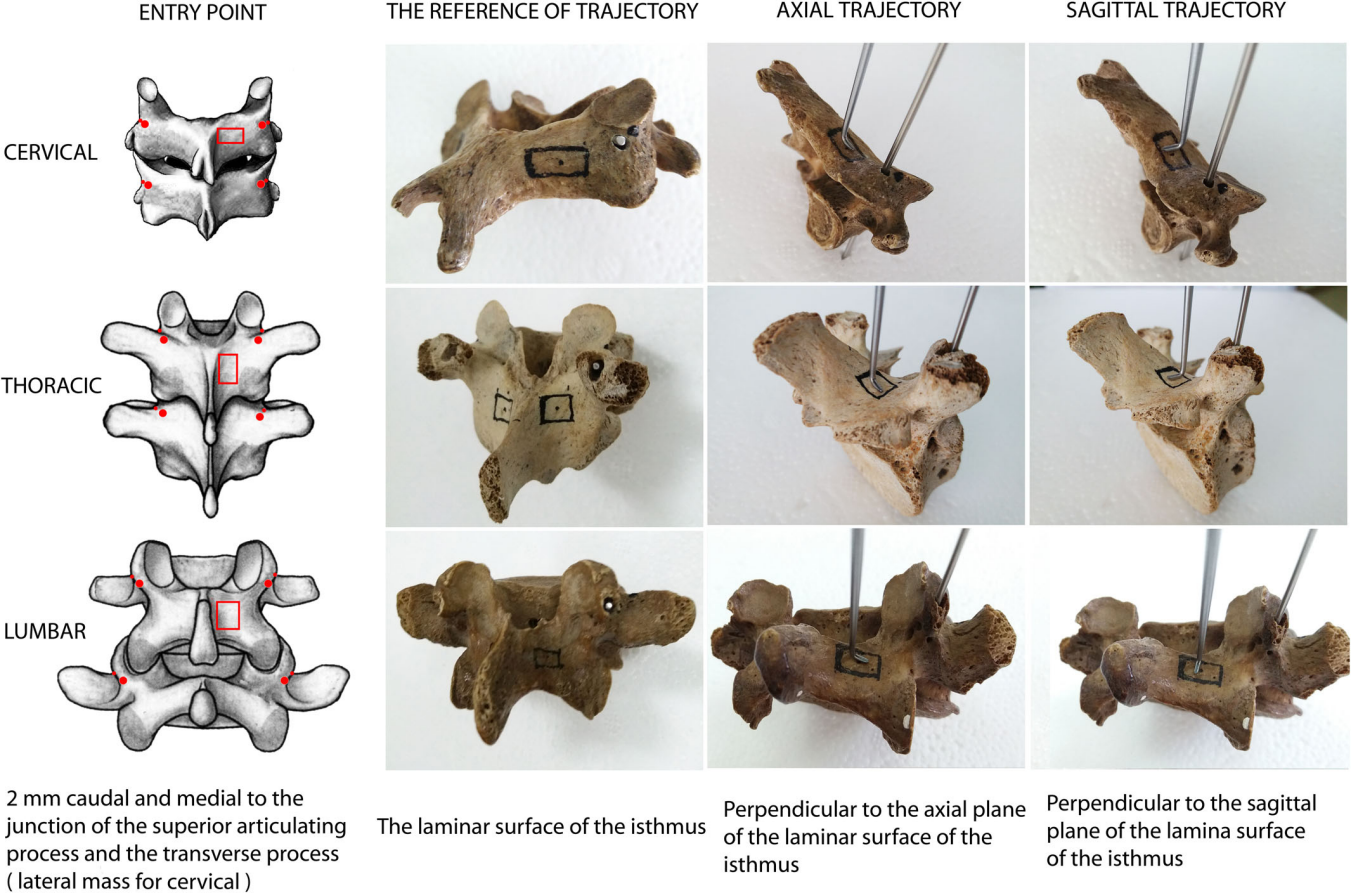
Illustration demonstrating the universal entry point and sagittal and axial trajectory for all subaxial cervical, thoracic and lumbar spine placements. The junction of the lateral margin of the superior articulating process and the transverse process for the thoracic and lumbar spine, or the lateral mass for the subaxial cervical spine, was determined. The entry point was at 1 mm, 2 mm, and 3 mm (2 mm in averaged) caudal and medial to this junction for the subaxial cervical, thoracic and lumbar spine, respectively (left column). Both sagittal and axial trajectories were perpendicular or orthogonal to the sagittal and axial planes of the laminae of the isthmus (right three columns).

The anatomic landmark of the junction of the lateral margin of the superior articulating process and the transverse process for thoracic and lumbosacral spines, or lateral mass for the subaxial cervical spine, was determined. The entry point was chosen at 1 mm, 2 mm, and 3 mm (2 mm on average) caudally and medially to this junction for the subaxial cervical, thoracic and lumbosacral spines, respectively (Fig. 1).

For determination of the frame of reference for sagittal and axial trajectories, the surface of the laminae of the isthmus was determined. The area of the four lines was determined to be as follows: below the lower edge of the superior articular process, above the upper edge of the inferior articular process, the medial edge of the articular process, and lateral edge of the spinous process (the medial edge of the laminae). Both sagittal and axial trajectories were perpendicular or orthogonal to the sagittal and axial planes of the laminae of the isthmus (Fig. 1).

### *Specimens*

This research has been approved by the IRB of the authorsʼ affiliated institutions. Two spine vertebrae specimens, including the segments from C_3_ to S_1_ vertebrae, were used for the present study. Both sides of each vertebra were inserted with a Gram needle 2.5 mm according to the entry point and sagittal and axial trajectory described above. After placing an iron scurf at the junction of the lateral margin of the superior articulating process and the transverse process (lateral mass for the subaxial cervical spine), the superior, lateral and axial of the Gram needle X‐ray films were obtained for verification of the trajectory.

### *3D CT Images*

3D reconstructions of spine CT images were obtained for 20 cases. A 3D model of every two vertebra from C_3_ to S_1_ were used to test the entry point and sagittal and axial trajectory described above. According to the vertebral anatomic structure, the entry point and surface of the laminae of the isthmus were determined with Hyperworks software (Altair, USA). After the planes that were orthogonal to the sagittal and axial surface of the laminae of the isthmus were established, the pedicle virtual pin tracts were determined. The 3D image was rotated, and the superior, lateral and axial views were obtained for verification of the trajectory.

### *Patients*

During an 8‐year period from January 2010 to December 2017, 610 consecutive patients underwent open pedicle screw placement in the subaxial cervical, thoracic and lumbosacral spines. In these 610 patients (412 male and 198 females), the aetiologic diagnosis included cervical trauma (27 cases), thoracolumbar trauma (180 cases), spinal tumor (135 cases), spinal deformity (145 cases), spinal infection (83 cases), and spinal degenerative diseases (43 cases); the distribution of the spine level was from C_3_ to S_2_, and the age ranged from 5 to 84 years.

### *Surgical Technique*

All cases were performed by the author (Z.Z.). All pedicle screws were inserted using the freehand technique. After exposure, a straight awl was used to disrupt the cortical bone at the entry point described above. A straight, blunt‐ended gearshift was used to cannulate the pedicle to the desired depth based on the sagittal and axial trajectory described above. A ball‐ended feeler was used to search for breaches. After the typical tapping and probing was repeated, an appropriate size screw was placed. To decrease the operative time, the markers were not used. Intraoperative fluoroscopy was used for initial localization and then again for a final anteroposterior and lateral radiograph.

### *Postoperative Computed Tomography Scans*

After the surgery and during the following up, standard anteroposterior and lateral films were taken to assess spinal injury, reduction, decompression, internal fixation, and fusion for all 610 patients. Among them, 68 patients underwent postoperative computed tomography (CT) scans, including 26 cervical cases, 19 scoliosis thoracic cases, 10 non‐scoliosis thoracic cases, eight lumbar cases, and five sacral cases. This study was based upon postoperative CT scans because CT scans have been shown to be more sensitive than radiographs in detecting cortical breaches^16^. The author does not routinely obtain postoperative CT scans on every patient undergoing fusion because of cost and added radiation exposure, but the author does perform CT scans if they are clinically indicated for proper purposes, such as planning for further surgery, evaluation of bony resection, baseline to follow tumor recurrence or progression, and radiosurgery treatment planning. The records of placements of pedicle screws of postoperative CT scans were retrospectively reviewed.

### *Classification of Pedicle Screw Positioning*

Placements of pedicle screws were assessed using CT data and outcome‐based classifications modified from previously reported systems as follows^15^, ^17^, ^18^, ^19^: Type 0 (good): screw within the pedicle medullary canal; Type 1 (acceptable): minimal penetration of the pedicle wall by the screw (<2 mm of medial or lateral cortical perforation or anterior cortex perforation); Type 2 (unacceptable): less than half of the diameter of the screw was outside the pedicle wall (2–4 mm of perforation); and Type 3 (grievous): more than half of the diameter of the screw was outside the pedicle wall (>4 mm of perforation) (spinal cord injury or screw abutting the aorta or vertebral artery). Types 2 and 3 were classified as grades of perforation or misplacements.

### *Statistical Analysis*

Statistical analyses were performed using SPSS13.0 software (SPSS Inc., Chicago, Illinois, USA) to evaluate the accuracy of screw placement. The placement of acceptable pedicle screws (Type 0 and 1) and unacceptable pedicle screws (Type 2 and 3) were compared using the Student's *t* test, with *P* < 0.05 considered to be statistically significant.

## Results

### *Trajectory Observation in Specimens and 3D Images*

**Figure 2:**
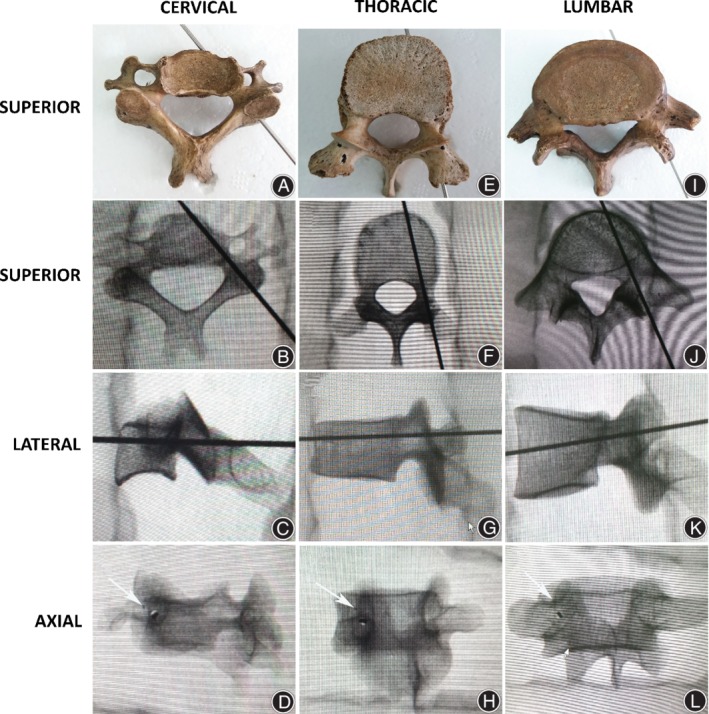
Trajectory observation after inserting a Gram needle for 2.5 mm according to the described technique of the entry point and sagittal and axial trajectory. The Gram needle is located in the center of pedicle of the superior, lateral and axial X‐ray views for cervical (A‐D), thoracic (E‐H), and lumbar (I‐L) spines. The junction of the lateral margin of the superior articulating process and the transverse process (lateral mass for subaxial cervical spine) is located at 11 o'clock of the pedicle's axial view (D, H, L) after placing an iron scurf at these junctions (arrows).

**Figure 3:**
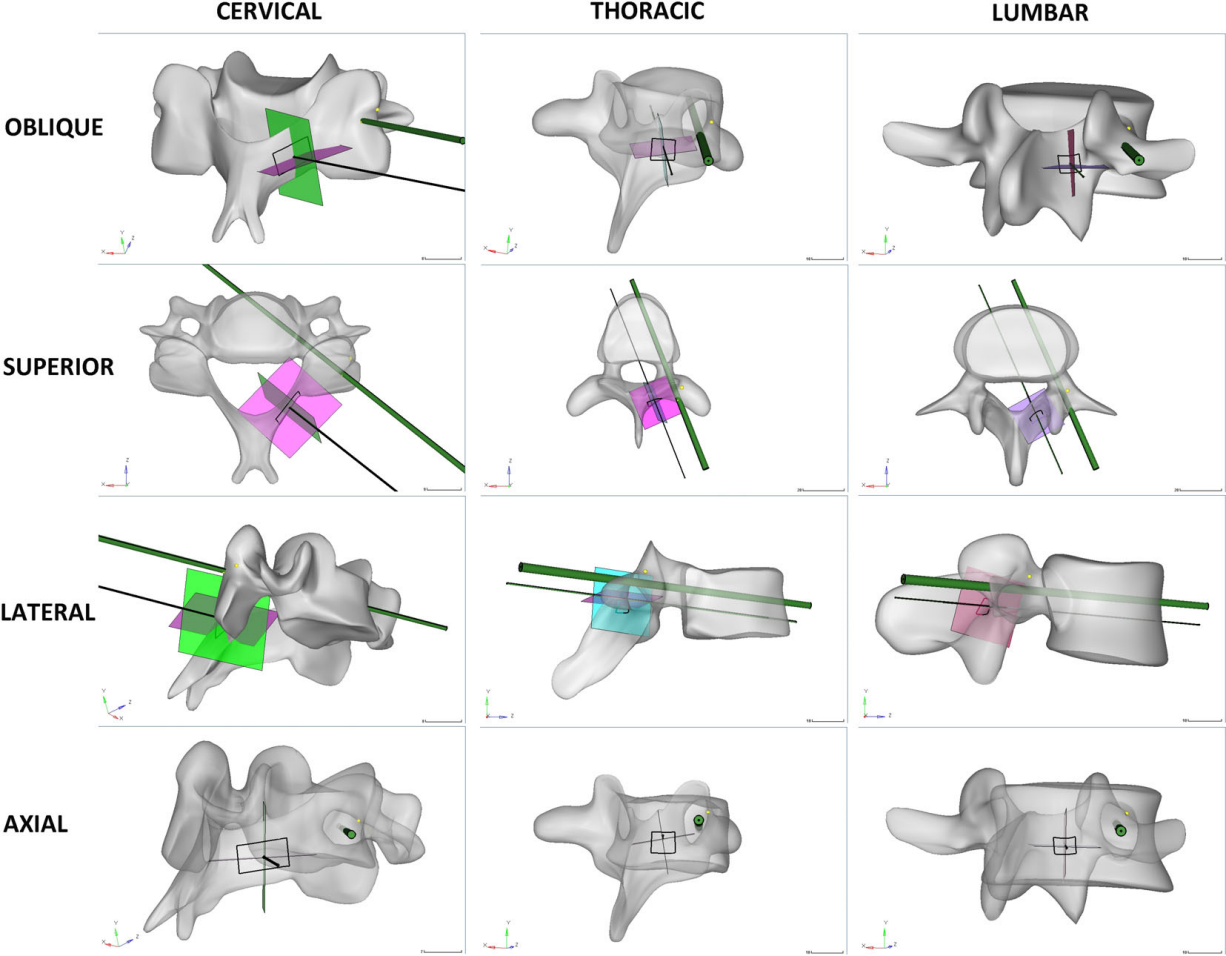
Trajectory observation after the pedicle virtual pin tracts were determined according to the described technique of the entry point and sagittal and axial trajectory. The pedicle virtual pin tractis located in the center of the pedicle of the superior, lateral, and axial views for cervical (left column), thoracic (middle column), and lumbar (right column) spines. The junction of the lateral margin of the superior articulating process and the transverse process (lateral mass for subaxial cervical spine) is located at 11 oʼclock of the pedicle's axial view (yellow spot).

After placing the iron scurf at the junction of the lateral margin of the superior articulating process and the transverse process (lateral mass for subaxial cervical spine), iron scurf was located at 1 o'clock or 11 o'clock of the pedicle's axial view (Fig. 2D, H, L). After inserting the 2.5 mm Gram needle or the pedicle virtual pin tracts according to the entry point and sagittal and axial trajectory described above, the Gram needle or the pedicle virtual pin was located in the center of the pedicle of the superior, lateral and axial views (Fig. 2), which indicated that the presented trajectory was located in the pedicle's axial trajectory. After the pedicle virtual pin tracts were determined according to the described technique of the entry point and sagittal and axial trajectory in 3D CT images, the pedicle virtual pin tract was located in the center of the pedicle of the superior, lateral and axial views for cervical, thoracic and lumbar spines (Fig. 3). The junction of the lateral margin of the superior articulating process and the transverse process (lateral mass for the subaxial cervical spine) was located at 11 o'clock of the pedicle's axial view (Fig. 3, yellow spot).

### *Clinical Accuracy*

**Figure 4:**
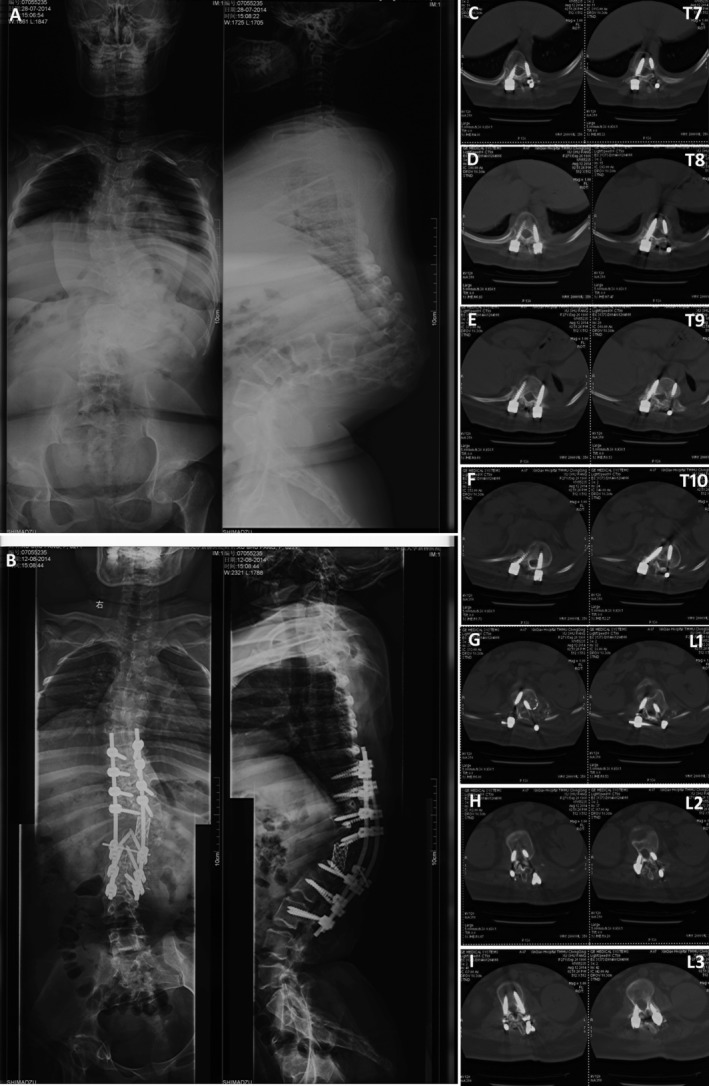
Postoperative computed tomography (CT) assessment of the pedicle screw position in a kyphoscoliosis patient according to the classification system: (A, B) pre‐ and postoperative X‐rays; (C) T7, right defined as grade II for medial, left as grade I for lateral; (D) T8, right defined as grade I for medial, left as grade 0; (E) T9, right defined as grade I for lateral, left as grade 0; (F) T10, right defined as grade 0, left as grade I for lateral; (G) L1, right defined as grade I for lateral, left as grade 0; (H) L2, right defined as grade 0, left as grade 0; (I) L3, right defined as grade 0, left as grade 0.

**Figure 5:**
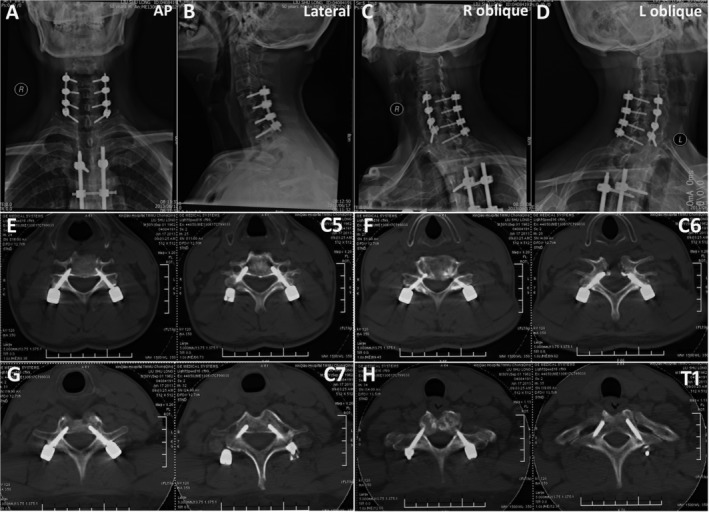
Postoperative computed tomography (CT) assessment of the pedicle screw position in a cervical infection patient according to the classification system: (A‐D) postoperative X‐rays; (E) C5, right defined as grade 0, left as grade 0; (F) C6, right defined asgrade 1 for medial, left as grade 1 for medial; (G) C7, right defined asgrade 0, left as grade 0; (F) T1, right defined asgrade 0, left as grade 0.

Among the 68 postoperative CT scan patients, including 21 kyphoscoliosis patients and 47 non‐kyphoscoliosis patients, a total of 766 pedicle screws were placed, including 316 screws in the kyphoscoliosis group and 450 screws in the non‐kyphoscoliosis group. It was found that 8% of the screws in the kyphoscoliosis group had penetrated the pedicle wall (Types 2 and 3) (Fig. 4), and 1% of the screws in the non‐kyphoscoliosis group penetrated the pedicle wall (Fig. 5), with almost the same rate of medial or lateral cortical perforation. A significant difference was observed between the acceptable (Types 0 and 1) and unacceptable (Types 2 and 3) pedicle screws’ accuracy in kyphoscoliosis group (*t* = 8.758, *P* = 0.000) and non‐kyphoscoliosis group (*t* = 9.985, *P* = 0.000) (Table 1). There were no screws with any neurological, vascular, or visceral complications in the 610 total patients.

**Table 1:** Distribution accuracy of pedicle screws placed at each level

Locations	Kyphoscoliosis group (21 cases)					Non‐kyphoscoliosis group (47 cases)				
	Acceptable		Unacceptable		Total	Acceptable		Unacceptable		Total
	T0	T1 (M/L)	T2 (M/L)	T3 (M/L)		T0	T1 (M/L)	T2 (M/L)	T3 (M/L)	
C_3_	3	1 (1/0)	0	0	4	17	2 (1/1)	0	0	20
C_4_	5	1 (0/1)	0	0	6	18	3 (2/1)	0	0	21
C_5_	4	0	0	0	4	27	3 (1/2)	1 (0/1)	0	31
C_6_	4	0	0	0	4	37	2 (1/1)	0	0	39
C_7_	3	0	1 (1/0)	0	4	39	4 (2/2)	0	0	43
T_1_	15	3 (2/1)	0	0	18	21	2 (0/2)	0	0	23
T_2_	15	3 (2/1)	0	1 (0/1)	19	26	2 (1/1)	1 (0/1)	0	29
T_3_	16	2 (0/2)	2 (1/1)	1 (0/1)	21	20	2 (0/2)	0	0	22
T_4_	15	2 (1/1)	2 (1/1)	0	19	23	2 (1/1)	0	0	25
T_5_	15	2 (1/1)	1(1/0)	0	17	17	2 (1/1)	0	0	19
T_6_	16	1 (0/1)	2(1/1)	1(0/1)	20	15	2 (2/0)	0	0	17
T_7_	18	3 (1/2)	2(1/1)	0	23	16	0	0	0	16
T_8_	15	4 (3/1)	1(1/0)	1(1/0)	21	16	0	0	0	14
T_9_	15	4 (2/2)	2(2/0)	1(1/0)	22	12	0	0	0	12
T_10_	14	2 (0/2)	2(1/1)	0	18	14	2 (1/1)	0	0	16
T_11_	11	1 (1/0)	2(1/1)	0	14	14	0	0	0	14
T_12_	12	1 (1/0)	1(0/1)	0	14	12	0	0	0	12
L_1_	10	2 (1/1)	2(0/2)	0	14	11	2 (0/2)	0	0	13
L_2_	17	1 (1/0)	0	0	18	11	2 (1/1)	0	0	13
L_3_	19	1 (1/0)	0	0	20	14	0	0	0	14
L_4_	7	1 (0/1)	0	0	8	12	0	0	0	12
L_5_	4	0	0	0	4	12	0	0	0	12
S_1_	2	0	0	0	2	7	0	0	0	7
S_2_	2	0	0	0	2	5	0	0	0	5
Total	256 (81%)	35 (18/17) (11%)	20 (11/9) (6%)	5 (2/3) (2%)	316	416 (92%)	32 (14/18) (7%)	2 (0/2) (1%)	0	450
	291 (92%)		25(8%)		316	448 (99%)		2 (1%)		450

L, lateral; M, medial; T, Type.

## Discussion

### *Embryological Evidence*

**Figure 6:**
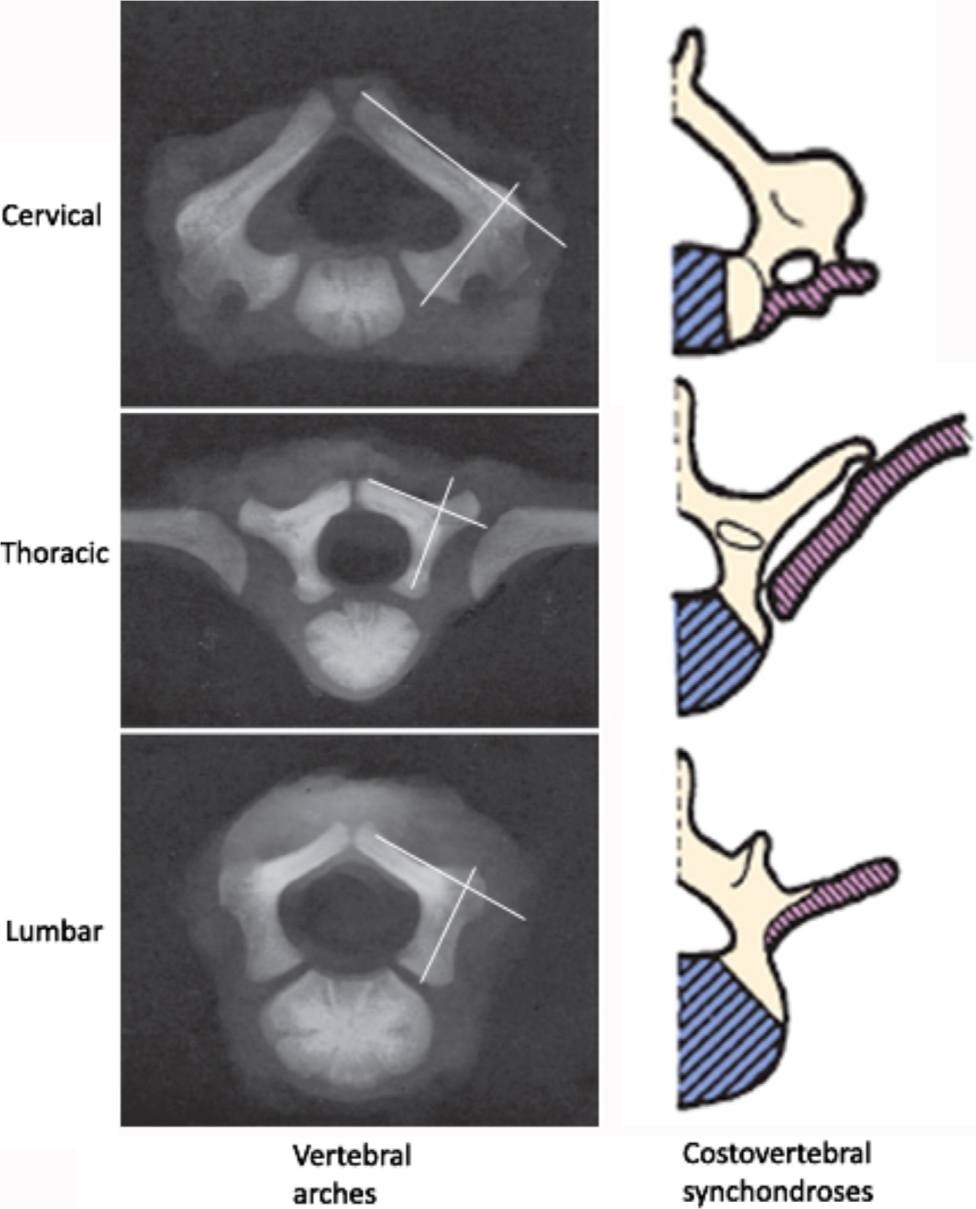
Development of the pedicles. The laminar of the isthmus and pedicle are developed from ossification of the same vertebral arches on a superior radiograph (left column). Costovertebral synchondroses developed a true rib and diarthrodial joint in the thoracic region, and the transverse process was observed in the lower cervical and lumbar vertebrae (right column).

The present choice of anatomic landmarks for the junction of the lateral margin of the superior articulating process, the transverse process or lateral mass, and the laminae of the isthmus was based on them developing from the same vertebral arches in the embryologic stage. There are three primary ossification centers in the typical embryonic vertebrae: one in the center of the centrum and one in each of the vertebra arch halves. The fusion of the fetal vertebral arches to the center occurs well anterior to the pedicles^20^. Therefore, the structure of vertebral arches, including the superior articulating process, the transverse process or lateral mass, and the laminae of the isthmus are theoretically anatomically fixed (Fig. 6). There are five secondary ossification centers: one in the tip of the spinous process, one in each transverse process tip, and one ring epiphysis in the superior and inferior endplates of the vertebral bodies^21^. These ossification centers may fuse troublesome structures during development, which are not reliable anatomic landmarks for pedicle insertion. The transverse process of the lower cervical vertebrae is developed from an additional costal center of ossification, which produces the true ribs and costovertebral joints for the thoracic spine^22^ (Fig. 6). This is the reason why we chose the lateral mass but not the transverse process as the anatomic landmark for the cervical spine.

### *Advantages*

The present trajectory is located in the pedicle's axial trajectory, which presented several advantages compared to various traditional freehand pedicle screw placement techniques (Table 2)^1^, ^2^, ^3^, ^4^, ^5^, ^6^, ^7^, ^8^, ^9^, ^10^, ^11^, ^12^, ^13^. First, it is a universal entry point throughout all subaxial cervical, thoracic and lumbosacral spine sections, which is easy to explore and identify without the influence of ossification. The present entry point is inherently located at 1 o'clock or 11 oʼclock of the pedicleʼs axial view, which may complement a surgeonʼs preference and/or bias for the familiar techniques. Second, it is a universal sagittal and axial plane technique that is orthogonal to the sagittal and axial plane of the laminae of the isthmus. This is the first report on the determination of sagittal and axial planes according to an anatomic landmark, which is easier to visualize intraoperatively than the anamnesis of traditional corresponding angulars for different levels. Third, it is also effective with kyphoscoliosis deformities. Because of the superior articulating process, the transverse process or lateral mass, and the laminae of the isthmus developed from the same vertebral arches in the embryologic stage, they are reliable anatomic landmarks even if a kyphoscoliosis deformity develops. Last, pedicle markers were not used in the present technique. Instead, the final anteroposterior and lateral radiographs were obtained after the screw had been placed intraoperatively, which obviated the need for yet another radiograph and a minor increase in operative time.

**Table 2:** The reported the methods and accuracy of free hand pedicle screw placement

Locations	Authors (Year)	Entry Point	Axial Trajectory	Sagittal Trajectory	Accuracy	
					Kyphoscoliosis	Non‐kyphoscoliosis
Cervical	Abumi, *et al*. (1994)^1^	Lateral to the center of the facet and close to the posterior margin of the superior articular surface.	Vary from 25° to 45° medial to the midline in the horizontal plane.	Parallel to the upper endplate of the vertebral body.	Hojo *et al*. (2014)^2^ reported 283 patients with 1065 screws. Overall malposition rate was 14.8%.	
	Ebraheim, *et al*. (1997)^3^	The superior and lateral corner of the lateral mass.	40°–47° laterally for C_3_‐C_6_, and 30°–40° laterally for C_7_.	10° cephalad for C6 and C_7_, 10° caudad for C_3_ and C_4_ and vertically for C_5_		
Thoracic	Kim, *et al*. (2004)^4^	T_1_‐T_2_: junction of the transverse process and lamina at the lateral pars interarticularis; T_3_‐T_6_: getting more lateral and caudal; T_7_‐T_9_: junction of proximal edge of the transverse process and lamina just lateral to the midportion of the base of the superior articular process; T_11_‐T_12_: junction of the transverse process and lamina or just medial to the lateral aspect of the pars interarticularis.	Proximal thoracic region: more lateral and caudal. Apical mid‐thoracic region: more medial and cephalad.		Etiologic diagnoses were: scoliosis in 273, kyphosis in 53, other spinal disease in 68. Total 3204 screws were inserted. Thirty‐six screws (6.2%) were inserted with moderate cortical perforation,	
	Modi, *et al*. (2009)^5^	The junction of the outer third and inner two‐thirds of the superior facet joint taken at the junction of the lateral and medial thirds of the facet joint after observing the whole facet joint margin.			Of the 854 inserted screws, the accuracy rate of screws inserted in the upper, middle and lower thoracic pedicles were 94.2, 91.6 and 93.7%, respectively.	
	Modi*, et al*. (2010)^6^	The junction of the outer third and inner two‐thirds of the superior facet joint taken at the junction of the lateral and medial thirds of the facet joint after observing the whole facet joint margin.			Of 482 inserted screws, the accuracy rate was 90.7%.	
	Parker, *et al*. (2011)^7^	The center of a triangular bony confluence formed by the superior articular facet, the transverse process, and the pars interarticularis. Occurs medial to the lateral margin of the superior articular process.	Medio‐lateral trajectory is performed to triangulate the screw insertion from lateral to medial.	Rostro‐caudal trajectory parallels the superior endplate of the segment of interest.	A total of 964 patients received 6816 screws in the thoracic or lumbar spine. The accuracy rate was 91%.	
	Fennell, *et al*. (2014)^8^	For each level: 3 mm caudal to the junction of the transverse process and the lateral margin of the superior articulating process.	Approximately 30° at T_1_ and T_2_, and 20° from T_3_ to T_12_.	Always orthogonal to the dorsal curvature of the spine at corresponding level.	A total of 219 thoracic pedicle screws were placed with a 96% accuracy rate.	
Lumbar	Magerl (1984)^9^	The junction of the lateral border of the superior articular process and the bisector of the transverse process	It is slightly oblique towards the midline (on an average 10°‐15°).	Checked preoperatively by various imaging studies such as CT scan or MRI.	Su *et al*. (2012)^10^ reported a total of 146 screw including 46 lumbar screws were inserted in scoliosis patients, with a 93.5% accuracy rate.	
	Roy‐Camille, *et al*. (1986)^11^	The intersection between the midlines of the facet joint and transverse process.	Straight on (0°).			Silbermann *et al*. (2011)^12^ reported a total of 30 patients received 152 screws with a 94.1% accuracy rate.
	Weinstein, *et al*. (1988)^13^	The inferior and lateral corner of the superior articular process, in the so‐called “nape of the neck.”	An average 10°–15° (L_1_, 5°; L_2_, 5°–10°; L_3_, 10°; L_4_, 10°–15°; L_5_, 15°).		Parker *et al*. (2011)^7^ reported a similar method in various spine disease. A total of 964 patients received 6816 screws including 3373 lumbar screws, with a 99.1% lumbar accuracy rate.	
Present study		The entry point was chosen at 1mm, 2mm, and 3mm (2 mm in averaged) of caudal and medial to the junction of the lateral margin of the superior articulating process and the transverse process (or lateral mass for subaxial cervical spine) for subaxial cervical, thoracic and lumbosacral spine, respectively.	orthogonal to the axial and sagittal plane of the laminar of isthmus		A total of 316 pedicle screws were placed with a 92% accuracy rate.	A total of 450 pedicle screws were placed with a 99% accuracy rate.

### *Clinical Accuracy*

Several studies have shown that all reported neurological deficits had medial pedicle perforations of more than 4 mm^17^, ^23^, ^24^, and no neurovascular complications have been reported with less than 2 mm medial perforation of the pedicle wall^25^, ^26^, ^27^. Although assessment of pedicle screws has been done with various classification systems, most of the authors stressed the fact that perforations that were less than 2 mm can be acceptable, not only regarding clinical safety but also on its biomechanical strength^28^, ^29^. After the postoperative assessment of the pedicle screw placements, the authors reported the relative accuracy rates for freehand techniques (Table 2)^1^, ^2^, ^3^, ^4^, ^5^, ^6^, ^7^, ^8^, ^9^, ^10^, ^11^, ^12^, ^13^. Generally, the overall accuracy rates were more than 90% for the thoracic and lumbar spine sections. There was no comparative study between kyphoscoliosis and non‐kyphoscoliosis groups based on the same technique. The medial perforation was more common than lateral perforation, and the percentage of misplaced screws in the thoracic spine was quite significant compared with the negligible complications observed. In the present technique, the relative accuracy rates were comparable with these studies. The non‐kyphoscoliosis group had a slightly higher accuracy rate than the kyphoscoliosis group, but there was no significant difference between the two groups. There were no neurovascular complications due to the use of this technique.

### *Recommendations*

We propose several recommendations regarding this technique. Preoperatively, pedicle CT data should be carefully identified, such as the diameter, no medullary cavity or an extremely small medullary cavity, and a developmental disturbance in neurofibromatosis‐1. Intraoperatively, because the present technique relies on pedicle cannulation, care is taken to appreciate the perforation of cancellous bone over the entire length of the pedicle and vertebral body. The blunt‐ended gearshift should advance smoothly with consistent resistance. Abrupt loss of resistance indicates a pedicle breach, while an increase in resistance indicates the gearshift is against the cortical bone, and the trajectory must be re‐evaluated prior to further advancement. During hand drilling, cannulating and tapping, the four walls and the floor of the pedicle track should be carefully probed, with particular attention being paid to the first 15–30 mm of the track and the lateral wall. The surgeon always retreats and repeats the palpation when an abnormal feeling is encountered to get sufficient information about the pedicle walls for decisions of adjustment or abortion. If there is any doubt on the final anteroposterior and lateral radiograph, the screw should be withdrawn, re‐probed, or adjusted if need be. For the lumbar spine, it does not need to be explored the junction of the lateral margin of the superior articulating process and the transverse process for the identification of the entry point, which may increase the bleeding and operation time. It is easy to detect this junction with a straight probe; the entry point is actually located on the superior articulating process. Finally, the assistance of a navigation system can improve the accuracy of pedicle placement, and it plays a key role in pedicles with no medullary cavity or an extremely small medullary cavity if there is high risk of failure of the present technique.

### *Limitations*

The present study has some limitations. First, this was a retrospective study with a relatively small sample size. Second, the data reported here were obtained by a single surgeon. Actually, the same technique was performed regularly by four senior surgeons in our department. They discontinued use of the traditional techniques, and no neurovascular complications were reported due to the use of this technique. The present study excluded these perioperative data. Finally, no cadaveric axial trajectory measurements were performed.

### *Conclusions*

It is feasible to employ freehand pedicle screw placement using the universal entry point and sagittal and axial trajectory for all subaxial cervical, thoracic and lumbosacral spines placements, and it is also effective with kyphoscoliosis deformities. While other techniques are effective and widely employed, this particular method may be easier to learn and adopt.
